# Serum angiopoietin-like 3 levels are elevated in obese non diabetic men but are unaffected during an oral glucose tolerance test

**DOI:** 10.1038/s41598-020-77961-8

**Published:** 2020-12-03

**Authors:** Maria Fernanda Garcés, Julieth Daniela Buell-Acosta, Haiver Antonio Rodríguez-Navarro, Estefania Pulido-Sánchez, Juan José Rincon-Ramírez, Diana Carolina Moreno-Ordóñez, Roberto Franco-Vega, Jhoan Sebastian Roncancio-Muñoz, Alvaro Javier Burgos-Cardenas, Ezequiel Lacunza, Justo P. Castaño, Carlos Diéguez, Rubén Nogueiras, Elizabeth Sanchez, Jorge Eduardo Caminos

**Affiliations:** 1 Department of Physiology, School of Medicine, Universidad Nacional de Colombia, Bogotá, Colombia; 2 Endocrine Unit - Department of Internal Medicine, School of Medicine, Universidad Nacional de Colombia, Bogotá, Colombia; 3 Department of Internal Medicine, School of Medicine, Universidad Nacional de Colombia, Bogotá, Colombia; 4 CINIBA, Facultad de Ciencias Médicas, Universidad Nacional de La Plata, La Plata, Argentina; 5 Maimonides Institute of Biomedical Research of Cordoba (IMIBIC), Córdoba, Spain; 6 Department of Cell Biology, Physiology and Immunology, Universidad de Córdoba, Córdoba, Spain; 7 Reina Sofia University Hospital, Córdoba, Spain; 8 CIBER Physiopathology of Obesity and Nutrition (CIBERobn), Córdoba, Spain; 9 Department of Physiology (CIMUS), School of Medicine, Instituto de Investigaciones Sanitarias (IDIS), Universidad de Santiago de Compostela, Santiago, Spain; 10 Department of Physiology, School of Medicine, Universidad Nacional de Colombia, Carrera 30 No. 45-03, Edificio 471 Piso 4 Oficina 406, Bogotá, Colombia

**Keywords:** Fat metabolism, Metabolic diseases

## Abstract

This study aimed to determine ANGPTL3 serum levels in healthy young lean and obese non-diabetic men during an oral glucose tolerance test (OGTT) and correlate them with anthropometric, biochemical and hormonal parameters. A case–control study was carried out and 30 young obese non-diabetic (23.90 ± 3.84 years and BMI 37.92 ± 4.85 kg/m^2^) and 28 age-matched healthy lean (24.56 ± 3.50 years and BMI of 22.10 ± 1.72 kg/m^2^) men were included in this study. The primary outcome measures were serum basal ANGPTL3 and ANGPTL3–area under the curve (AUC) levels. The percentage of body fat was measured by dual-energy X-ray absorptiometry and biochemical, hormonal and insulin resistance indices were determined. Basal ANGPTL3 and ANGPTL3–AUC levels were significantly elevated (p < 0.05) in young obese subjects compared with lean subjects and were positively and significantly associated with different anthropometric measurements. Fasting ANGPTL3 serum levels were positively correlated with fasting insulin, leptin, Leptin/Adiponectin index and triglyceride—glucose index. Moreover, ANGPTL3–AUC was negatively correlated with Matsuda index. In this regard, chronically high ANGPTL3 levels in young obese subjects might favor triglyceride-rich lipoprotein clearance to replenish triglyceride stores by white adipose tissue rather than oxidative tissues.

## Introduction

Elevated plasma triglyceride levels are associated with increased risk of atherosclerosis and cardiovascular disease, a major cause of morbidity and mortality worldwide^1^. Lipoprotein lipase (LPL) plays a fundamental role in the normal lipid metabolism and energy balance, by catalyzing the hydrolysis of the triglyceride (TG) component of circulating chylomicrons and very-low-density lipoprotein (VLDL) at the luminal surface of endothelial cells in extrahepatic tissues, to release fatty acids that can be used or stored^2–4^. LPL is mainly expressed in adipose tissue and striated muscle (skeletal and cardiac) and its activity is regulated in a tissue-specific manner^5,6^.

Different members of the angiopoietin-like (ANGPTL) family of proteins participate in the regulation of LPL activity, including ANGPTL3, ANGPTL4 and ANGPTL8^7,8^. ANGPTL3 is a secreted glycoprotein mainly expressed in the liver and acts as a negative regulator of LPL and endothelial lipase activity in oxidative tissues such as brown adipose tissue (BAT) and skeletal muscle^9^. Elevated circulating levels of this hepatokine are known to increase plasma levels of triglycerides, high-density lipoprotein cholesterol (HDL-c) and low-density lipoprotein cholesterol (LDL-c)^10^. In addition, Muniyappa et al., reported higher plasmatic levels of ANGPTL3 in leptin-deficient patients with lipodystrophy when compared to healthy control subjects^11^. Furthermore, leptin replacement therapy (metreleptin) in patients with lipodystrophy decreased significantly serum ANGPTL3 levels, total cholesterol and triglyceride concentrations^11^.

Consistent with clinical data, preclinical studies showed that the overexpression of both ANGPTL3 and ANGPTL8 in mice resulted in hypertriglyceridemia^12^. Moreover, plasma levels and hepatic gene expression of ANGPTL3 are increased in leptin-deficient *ob/ob* and leptin-resistant *db/db* mice and were associated with an increase in plasma triglycerides and free fatty acids^13^. Additionally, leptin-deficient *ob/ob* mice supplemented with recombinant leptin, showed lower gene expression and plasmatic levels of ANGPTL3 and normalization of plasmatic triglyceride levels^13^.

Despite ANGPTL3 was proposed as an important regulator of plasmatic lipoprotein metabolism through LPL inhibition, to date, contradictory results have been reported on ANGPTL3 circulating levels in obese subjects^2,3,14–16^. Furthermore, some studies have shown that ANGPTL3 can be involved in carbohydrate metabolism, but the evaluation of ANGPTL3 response to oral glucose stimulation has not been clearly established yet^2,17–20^. Therefore, the aim of this case–control study was to determine serum ANGPTL3 levels in young healthy lean and obese non-diabetic men in response to the oral glucose tolerance test (OGTT), a physiological imitator of meal stimulation. In addition, we studied the relationship between serum ANGPTL3 levels with anthropometric, biochemical, hormonal and insulin resistance indices.

## Materials and methods

### Ethical aspects

All experimental protocols were reviewed and approved by the Ethics Review Board of the School of Medicine of the Universidad Nacional de Colombia (permission number 012-204-18, August 27—2018) and a written informed consent was obtained from all study participants. All protocols were performed in accordance with the Declaration of Helsinki for Medical Research involving Human Subjects and approved methods.

### Study population

The present investigation was conducted as a case–control study at the Department of Internal Medicine—Division of Endocrinology School of Medicine Universidad Nacional de Colombia. According to the World health organization (WHO) body mass index (BMI = kg/m^2^) criteria, 30 young non-diabetic obese men (BMI ≥ 30 kg/m^2^) (23.90 ± 3.84 years and BMI 37.92 ± 4.85 kg/m^2^) and 28 young healthy lean men (BMI 18.50–24.99 kg/m^2^) (24.56 ± 3.50 years and BMI of 22.10 ± 1.72 kg/m^2^) were included in this study^4^. Healthy lean individuals in the study were normotensive, euglycemic and with triglycerides and cholesterol levels within the normal range^21^.

Subjects who met any of the following criteria were excluded from the study: current smoking, alcoholism, mental illness, subjects diagnosed with type 2 diabetes mellitus (T2DM), chronic kidney disease, cardiac failure, hepatic failure, thyroid diseases, infectious diseases among other diseases, use of approved weight lowering pharmacotherapy or patients with a history of gastric bypass and other bariatric surgery. Also, subjects who had taken drugs that affect energy metabolism, such as metformin, levothyroxine or steroids, within the last 12 months, were excluded.

### Clinical evaluation

Subjects underwent clinical and physical examination. Weight and height were determined and BMI (kg/m^2^) was calculated. Anthropometric data included height, hip circumference (HC), waist circumference (WC) and estimated measuring of body fat percent (BF%) was determined by Dual-Energy X-ray absorptiometry (DXA) (GE Lunar Prodigy Advance). Systolic blood pressure (SBP) and diastolic blood pressure (DBP) were measured in all subjects and mean arterial pressure (MAP) were calculated.

### Biochemical and hormonal analysis

All experiments started in the morning between 7:00 and 9:00 AM after an overnight fast. All subjects were given 75 g of anhydrous glucose, dissolved in 200 mL of water, after overnight fasting. The subjects underwent an oral glucose tolerance test (OGTT) and blood samples were collected at fasting and 30, 60 and 120 min after glucose ingestion. The serum samples were separated by centrifugation at 1000×*g* for 15 min and stored at − 80 °C until the respective assays. Serum levels of glucose (basal and postprandial), total cholesterol (TC), low-density lipoprotein cholesterol (LDL-c), high-density lipoprotein cholesterol (HDL-c), high-sensitivity C-reactive protein (hs-CRP) and triglycerides (TG) were determined. Additionally, serum levels of ANGPTL3 (basal and postprandial), serum insulin levels (basal and postprandial), serum levels of adiponectin and leptin, were analyzed.

Serum ANGPTL3 levels were measured using a commercial ELISA kit (Catalog Number DANL30—R&D Systems, Inc. USA). The intra- and inter-assay coefficients of variation (CVs) were < 4.1% and < 8.5%, respectively. Serum human leptin levels were measured with a commercial ELISA kit (Catalog Number KAC2281-ThermoFisher Scientic Inc.). The intra- and inter-assay coefficients of variation for leptin concentrations were < 3.9% and < 5.3%, respectively. Likewise, serum levels of adiponectin were determined by an ELISA KIT (Catalog Number KHP0041-ThermoFisher Scientific Inc.) and the coefficients of intra- and inter-assay variation were < 3.8% and < 5.5% respectively. All samples were analyzed in duplicates. Insulin–Area Under the Curve (AUC) (µUI–mL/2 h), glucose–Area Under the Curve (AUC) (mg–dL/2 h) and ANGPTL3–Area Under the Curve (AUC) (ng–mL/2 h) during OGTT were calculated by trapezoidal approximation rule as described elsewhere^22,23^.

### Definitions

All subjects underwent a full physical examination and measurements of anthropometric parameters were performed with a standardized technique. Anthropometric measurements were taken while the participants dressed in light clothing, without shoes. Body Mass Index (BMI = kg/m^2^) was calculated as weight (kg) divided by height (m) squared. Furthermore, we calculated the waist circumference (WC) to height ratio (WHtR), an anthropometric index for central adiposity, determined by dividing the WC by height (cm)^24^. Additionally, we measured the waist-to-hip ratio (WHipR) as described elsewhere^21^.

Metabolic syndrome was defined according to the International Diabetes Federation (IDF) criteria, to which central obesity (defined by WC) and ethnicity is an essential component for diagnosis of the syndrome and required the presence of two of the following elements: fasting plasma glucose (FPG) ≥ 100 mg/dL, systolic blood pressure (SBP) ≥ 130 mm Hg and diastolic blood pressure (DBP) ≥ 85 mm Hg, or on antihypertensive medication, TG ≥ 150 mg/dL or on treatment, HDLc < 40 mg/dL in men and < 50 mg/dL in women, or on treatment^21^. Additionally, borderline metabolic abnormalities for fasting glucose and high normal blood pressure (BP), as described by Baden et al.^25^. Moreover, borderline lipid profile for metabolic syndrome factor, among these, TC, LDLc, HDLc and TG are described elsewhere^26^.

Insulin resistance was determined according to the homeostatic insulin resistance assessment model (HOMA-IR) as described by the Matthews et al.^27^. Likewise, the quantitative insulin sensitivity check index (QUICKI) and Matsuda index for hepatic and muscle resistance/sensitivity were estimated as described elsewhere^28–30^. Triglyceride-glucose (TyG) and TG/HDL-c indices were calculated as described elsewhere^31,32^. Values of the leptin/adiponectin ratio (LAR) were made as Finucane et al. described in individuals with non-diabetic insulin resistance^33^. Leptin resistance is a term commonly used to define states in which hyperleptinemia, as seen in obesity-related condition, results in a decreased response to leptin^34^.

### Statistical analysis

Descriptive data were presented as mean ± SD. Variables with normal distribution were compared by unpaired Student’s t-test and one-way ANOVA and repeated-measured ANOVA. Additionally, a post hoc analysis was made among the groups. Mann–Whitney U test, Kruskal–Wallis one-way analysis of variance and Friedman test were performed for non-normal distribution variables. Pearson’s correlation coefficient was used to assess of linear relationship between fasting ANGPTL3 ng/mL and ANGPTL3–AUC (ng–mL/2 h) with different metabolic, anthropometrics and hormonal variables. A p value < 0.05 was considered to be statistically significant (*p-value < 0.05, **p-value < 0.01, ***p-value < 0.001). All statistical analyses were done using R version 3.4.0. Software^35^. Line plots and Boxplots were generated with the R BiocGenerics package^36^.

## Results

### Experimental results

**Figure 1:**
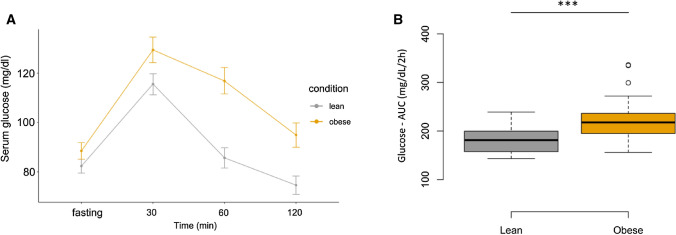
Comparison between serum glucose levels in the obese group and lean group: (**A**) at fasting, 30, 60, and 120 min and (**B**) serum glucose levels area under the curve (AUC) (mg/dL/2 h) in the obese group and lean group. Line plots and Boxplots were generated with the R BiocGenerics package. A p-value < 0.05, was considered statistically significant *p-value < 0.05, **p-value < 0.01, ***p-value < 0.001.

**Figure 2:**
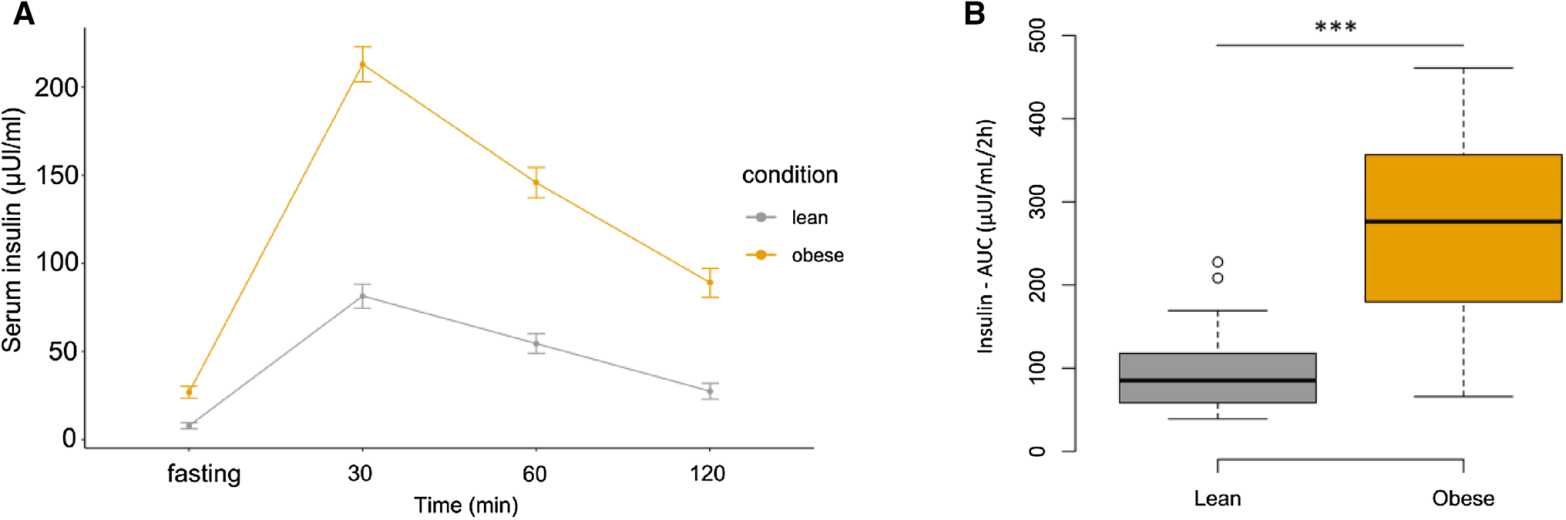
Comparison between serum insulin levels in the obese group and lean group: (**A**) at fasting, 30, 60, and 120 min and (**B**) serum insulin levels area under the curve (AUC) (μUI/mL/2 h) in the obese group and lean group. Line plots and Boxplots were generated with the R BiocGenerics package. A p-value < 0.05, was considered statistically significant *p-value < 0.05, **p-value < 0.01, ***p-value < 0.001.

Characteristics of the study population are shown in Table 1. Young obese subjects showed a borderline increase of SBP, DBP and MAP^25^. Additionally, obese subjects presented borderline risk factors for metabolic syndrome, including TC, LDLc, HDLc and TG^26^. During the OGTT, serum glucose and insulin levels were significantly higher in the obese group compare with lean group at fasting, 30, 60, and 120 min (Figs. 1, 2 respectively, Table 2). Glucose—AUC and insulin—AUC were significantly higher in obese compared with lean subjects (Figs. 1, 2, Table 2). Hyperinsulinemia and hyperleptinemia were confirmed in the obese subjects by the surrogated indices HOMA-IR, QUICKI, Matsuda, TyG, TG/HDL-c and LAR indices (Table 1).

**Table 1:** Baseline characteristics of the study population.

Variable	Lean (n = 28) Mean ± SD	Obese (n = 30) Mean ± SD	p value
Age (year)	24.56 ± 3.50	23.89 ± 3.88	0.480
Systolic blood pressure (SBP)	112.11 ± 10.18	129.43 ± 12.12	0.001
Diastolic blood pressure (DBP)	70.14 ± 8.10	85.03 ± 10.44	0.001
Mean arterial pressure (MAP)	84.12 ± 7.21	100.03 ± 9.71	0.001
Height (m)	1.74 ± 0.054	1.75 ± .0.065	0.412
Weight (kg)	66.79 ± 6.59	115.70 ± 15.75	0.001
BMI (kg/m^2^)	22.10 ± 1.72	37.92 ± 4.85	0.001
Hip (cm)	94.96 ± 3.58	123.02 ± 7.75	0.001
WC (m)	78.11 ± 4.60	110.99 ± 8.36	0.001
AFM (%)	28.93 ± 6.92	55.71 ± 3.14	0.001
GFM (%)	27.30 ± 4.72	47.37 ± 3.78	0.001
TBF (%)	14.44 ± 3.52	45.10 ± 3.70	0.001
WHtR	0.45 ± 0.03	0.63 ± 0.057	0.001
WhipR	0.82 ± 0.035	0.90 ± 0.054	0.001
Leptin (ng/mL)	7.54 ± 1.03	23.43 ± 7.76	0.001
Adiponectin (µg/mL)	15.14 ± 1.92	13.16 ± 1.79	0.001
Fasting glucose (mg/dL)	82.39 ± 7.97	88.53 ± 11.23	0.031
Fasting Insulin (µUI/mL)	7.83 ± 3.16	26.80 ± 11.51	0.001
Triglycerides (mg/dL)	91.75 ± 30.10	191.70 ± 98.48	0.001
HDLc cholesterol (mg/dL)	47.86 ± 7.09	40.37 ± 7.46	0.002
LDLc cholesterol (mg/dL)	98.86 ± 20.22	116.07 ± 22.82	0.003
Total cholesterol (mg/dL)	164.57 ± 23.97	189.30 ± 27.65	0.002
(hs-CRP)	1.01 ± 0.91	5.88 ± 4.15	0.001
Leptin/Adiponectin Index	0.51 ± 0.10	1.81 ± 0.63	0.001
TG/HDLc Index	1.99 ± 0.81	4.81 ± 2.63	0.001
TyG Index	8.16 ± 0.33	8.92 ± 0.48	0.001
HOMA–R Index	1.59 ± 0.64	6.41 ± 3.13	0.001
Matsuda Index	6.72 ± 2.86	2.03 ± 1.34	0.001
QUICKI Index	0.36 ± 0.023	0.30 ± 0.022	0.001

*AFM* android fat mass (%), *AUC* area under the curve, *BMI (kg/m*^*2*^*)* Body Mass Index, *DBP* diastolic blood pressure, *GFM* gynoid fat mass (%), *HDL-c* high-density lipoprotein cholesterol, *HOMA-IR* homeostatic insulin resistance assessment model, *hs-CRP* high-sensitivity C-reactive protein, *L/A* leptin/adiponectin ratio, *LDL-c* low-density lipoprotein cholesterol, *MAP* mean arterial pressure, *QUICKI* quantitative insulin sensitivity check index, *SBP*: systolic blood pressure, *TBF* total body fat (%), *TG* triglycerides, *TG/HDL-c* triglyceride (TG)/high-density lipoprotein cholesterol (HDL-c) (TG/HDL-c ratio) index, *TyG* triglyceride-glucose index, *VLDL-c* very low-density lipoprotein cholesterol, *WAT* white adipose tissue, *WC* waist circumference, *WHipR* waist-to-hip ratio, *WHtR* waist circumference to height ratio.

*A p-value < 0.05 was considered statistically significant.

**Table 2:** Comparison of glucose, insulin and ANGPTL3 serum levels at fasting, 30, 60 and 120 min between the lean and obese group.

Variable	Time	Lean (n = 28) Mean ± SD	Obese (n = 30) Mean ± SD	*p* value
Glucose	0 min (mg/dL)	82.39 ± 7.97	88.53 ± 11.23	0.031
	30 min (mg/dL)	115.61 ± 18.31	129.43 ± 26.68	0.027
	60 min (mg/dL)	85.71 ± 16.91	116.83 ± 28.83	0.001
	120 min (mg/dL)	74.64 ± 13.81	94.97 ± 24.12	0.001
	Glucose–AUC	180.01 ± 24.82	221.96 ± 44.02	0.000
Insulin	0 min (µUI/mL)	7.83 ± 3.16	26.80 ± 11.51	0.001
	30 min (µUI/mL)	81.46 ± 4.58	212.94 ± 9.75	0.001
	60 min (µUI/mL)	54.49 ± 30.82	145.77 ± 74.07	0.001
	120 min (µUI/mL)	27.46 ± 20.63	89.10 ± 68.47	0.005
	Insulin–AUC	88.21 ± 32.21	267.05 ± 108.90	0.000
ANGPTL3	0 min (ng/mL)	117.20 ± 24.05	140.09 ± 33.07	0.008
	30 min (ng/mL)	117.29 ± 23.90	135.6081 ± 29.87	0.013
	60 min (ng/mL)	113.24 ± 25.19	141.7284 ± 31.76	0.003
	120 min (ng/mL)	113.19 ± 31.85	142.5383 ± 30.81	0.002
	ANGPTL3–AUC	229.48 ± 46.92	280.39 ± 55.72	0.000

*AUC* area under the curve.

*A p-value < 0.05 was considered statistically significant.

**Figure 3:**
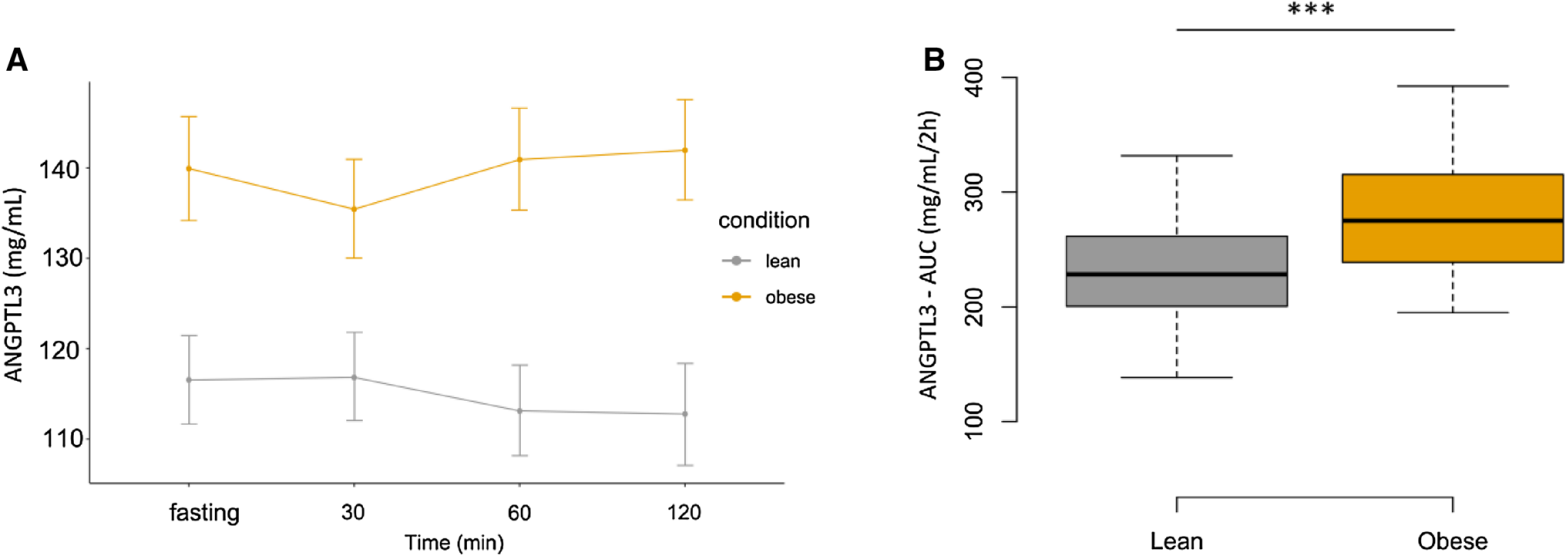
Comparison between serum ANGPTL3 levels in the obese group and lean group: (**A**) at fasting, 30, 60, and 120 min and (**B**) serum ANGPTL3 levels area under the curve (AUC) (ng/mL/2 h) in the obese group and lean group. Line plots and Boxplots were generated with the R BiocGenerics package. A p-value < 0.05, was considered statistically significant *p-value < 0.05, **p-value < 0.01, ***p-value < 0.001.

Serum adiponectin levels were significantly lower (p = 0.001) in obese non-diabetic men compared to the healthy lean men (Table 1). In contrast, serum leptin concentrations were notably higher (p = 0.001) in obese non-diabetic men compared to the healthy lean men (Table 1). Mean ANGPTL3 serum levels at 0, 30, 60, and 120 min and ANGPTL3–AUC, in response to an oral glucose tolerance test, were significantly higher (p = 0.000) in obese compared to healthy lean subjects (Table 2, Fig. 3). No significant difference was seen over time in serum ANGPTL3 levels at baseline (0), 30, 60 and 120 min during the OGTT in healthy young lean and obese non-diabetic men (Fig. 3).

At baseline, ANGPTL3 serum levels were significantly (but weakly) positively correlated with fasting insulin (r = 0.267; p = 0.043) (Table 3). Furthermore, leptin (r = 0.381; p = 0.003), LAR (r = 0.381; p = 0.003) and TyG indices (r = 0.285; p = 0.030) were positively correlated with fasting ANGPTL3 serum levels (Table 3, Supplementary Fig. S1A–C respectively). Fasting serum ANGPTL3 levels were positively correlated with anthropometric parameters such as BMI (r = 0.413; p = 0.001), WC (r = 0.453; p = 0.000), WHtR (r = 0.435; p = 0.001), AFM (%) (r = 0.395; p = 0.002), TBF (%) (r = 0.395; p = 0.002), WHipR (r = 0.417; p = 0.001) and GFM (%) (r = 0.350; p = 0.007) (Table 3). There was no statistically significant correlation between basal serum ANGPTL3 levels and triglyceride levels, HOMA–IR and QUICKI indices (Table 3).

**Table 3:** Correlation between basal ANGPTL3 serum levels and anthropometric, biochemical variables and insulin resistance indices.

Variable	Basal ANGPTL3		ANGPTL3–AUC	
	r	*p* value	r	*p* value
Weight (kg)	0.411	0.001	0.451	0.000
BMI (kg/m^2^)	0.413	0.001	0.417	0.001
Hip (cm)	0.392	0.002	0.443	0.000
WC (m)	0.453	0.000	0.459	0.000
AFM (%)	0.395	0.002	0.476	0.000
GFM (%)	0.350	0.007	0.428	0.000
TBF (%)	0.395	0.002	0.460	0.000
WTHR	0.435	0.001	0.418	0.001
WHipR	0.417	0.001	0.339	0.009
Leptin (ng/mL)	0.381	0.003		
Adiponectin (µg/mL)	− 0.133	0.321		
Fasting insulin (µUI/mL)	0.267	0.043		
Triglycerides (mg/dL)	0.176	0.187		
L/A ratio	0.381	0.003		
TyG index	0.285	0.030		
HOMA–R Index	0.234	0.077		
QUICKI Index	− 0.224	0.091		
Insulin–AUC			0.324	0.013
Matsuda Index			− 0.315	0.016

*AFM* android fat mass (%), *AUC* area under the curve, *BMI (kg/m*^*2*^*)* Body Mass Index, *GFM* gynoid fat mass (%), *TBF* total body fat (%), *TyG* triglyceride-glucose index, *hs-CRP* high-sensitivity C-reactive protein, *WC* waist circumference, *WHipR* waist-to-hip ratio, *WHtR* waist circumference to height ratio.

*A p-value < 0.05 was considered statistically significant.

Finally, ANGPTL3-AUC was negatively correlated with Matsuda Index (r =  − 0.315; p = 0.016) and positively correlated with Insulin-AUC (r = 0.324; p = 0.013) (Supplementary Fig. S3A,B respectively). Similar correlations between ANGPTL3–AUC and anthropometric parameters such as BMI (r = 0.417; p = 0.001), WC (r = 0.459; p = 0.000), WHtR (r = 0.418; p = 0.001), WHipR (r = 0.418; p = 0.009), AFM (%) (r = 0.476; p = 0.000), GFM (%) (r = 0.428; p = 0.000) and TBF (%) (r = 0.460; p = 0.000) (Supplementary Fig. S2A–D,F respectively), were seen with fasting ANGPTL3 serum levels (Table 3).

## Discussion

This study has demonstrated that serum ANGPTL3 levels were significantly elevated in young obese non-diabetic subjects compared to healthy control subjects. However, short‐term changes of serum ANGPTL3 levels at fasting and OGTT (30, 60, and 120 min) remained unchanged in both groups. Thus, the present study suggests that serum ANGPTL3 levels do not seem to be involved in short-term regulation of carbohydrate metabolism. However, previous studies have shown that ANGPTL3 levels are abnormally increased in insulin-resistant state and obesity, with worsening glucose metabolism and enhancing lipolysis in adipose tissues^19,37^. This could indicate the possible role of ANGPTL3 in regulating glucose metabolism at long-term, which could progressively lead to type 2 diabetes and other metabolic lifelong diseases^19,37^.

Basal serum ANGPTL3 levels were positively correlated with anthropometric parameters such as WC, BMI, WHipR, TBF (%), AFM (%) and GFM (%). However we did not find a significantly correlation with TG levels, which is in line with previously reported results^3,14^, but not with others showing that circulating ANGPTL3 was negatively correlated with TG levels^38^. The controversial relationship between ANGPTL3 levels and TG suggests that there are other factors affecting the interaction between ANGPTL3 levels and TGs, and further studies are needed to better understand this relationship^3,14,38,39^. In this regard, it is worth pointing out that ANGPTL3 levels are significantly higher in patients with advanced forms of NAFLD or nonalcoholic steatohepatitis (NASH), but no significant differences were detected in individuals with simple fatty liver (NAFL) when compared to healthy and normal weight individuals^20^. Thus, the results obtained in our study showing that ANGPTL3 levels were significantly higher in young obese non-diabetic individuals and could be at least partially, affected by the presence of NASH. This is relevant because the development of NASH reduces insulin clearance and could hinder the interpretation of plasma insulin data and derived indices.

Additionally, in this study, results showed serum ANGPTL3 levels at baseline and ANGPTL3–AUC significantly higher in obese subjects compared to lean subjects. These data are in agreement with previous results reported in human obese subjects and animal models^2,13,17^. In addition, plasmatic levels of ANGPTL3 were elevated in patients with generalized lipodystrophy and significantly reduced after metreleptin therapy^11^. Our data indicating that fasting serum ANGPTL3 levels were positively correlated with serum leptin levels and leptin/adiponectin index would suggest that hyperleptinemia might lead to increased serum ANGPTL3 levels in young obese subjects. However, different to ours, Cinkajzlová et al. have found serum ANGPTL3 levels were lower in obese subjects with and without T2DM when compared to healthy control subjects^16^. It is important to highlight that those results were obtained in obese subjects with metabolic syndrome, while our cohort showed borderline metabolic syndrome criteria risk factors. On the other hand, other studies demonstrated that ANGPTL3 levels did not differ significantly between overweight or obese subjects compared to normal weight subjects^14,15^.

It is important to highlight that Shimamura et al. also demonstrated the negative regulation of ANGPTL3 by insulin action^13^. In STZ-treated insulin-deficient mice, plasmatic levels and hepatic expression of ANGPTL3, TG and FFA levels were higher compared with control mice^13^. Of note, in the current study we observed ANGPTL3–AUC was positively correlated with insulin–AUC and negatively correlated with the Matsuda index, which would imply that insulin resistance might contribute to the increased serum ANGPTL3 levels in obese subjects. On the other hand, the associations of basal serum levels of ANGPTL3 with fasting insulin levels and TyG index was found to be weak as evidenced by their low correlation coefficients which were smaller than 0.29. Therefore, it is evident that assessing ANGPTL3 in obese subjects will require further investigation, in particular performing large and well conducted clinical and epidemiological studies, stratified by age, sex, ethnicity and geographic region.

Recent Genome-Wide Association Studies (GWAS) and whole-exome sequencing have identified sequence variants associated with loss of function in the ANGPTL3 gene across populations from Europe, Asia, Africa and the Americas^40–46^. In this way, ANGPTL3 genetic variation across populations from Europe, Asia, Africa and the Americas, has been studied to identify Single Nucleotide Polymorphism (SNPs) specific to each population. Therefore, sequence variant in the ANGPTL3 gene might have important implications for the diagnosis and therapeutic interventions of diseases such as combined hypolipidemia that consists of extremely low plasma levels of HDL-c, LDL cholesterol and triglycerides^40–46^. From a genetic perspective on diversity, the population of Latin American is descended from indigenous American, European, and African populations. SNPs and structural variants that may be unique or specific to each population for ANGPTL3 have not been studied extensively and it may have important consequences for health and wellbeing.

This paper is the first study of ANGPTL3 status in young obese non-diabetic and age-matched healthy non-obese men control in the Latino American population. However, a limitation of the study is that the possible prevalence of NAFLD in obese subjects, which may have an important influence in the ANGPTL3 levels that was not considered. In addition, only young men were enrolled in the study, with the purpose to reduce the effects of possible confounding variables, such as the influences of sex hormones in healthy women during normal menstrual cycle and in obese women with polycystic ovary syndrome (PCOS).

## Conclusions

In conclusion, our results indicate that in Latin American young obese individuals, circulating ANGPTL3 levels are higher than those non-obese healthy controls at the basal state, but these levels remained unchanged during an OGTT. Thus, these findings support an important role of ANGPTL3 in obesity.

## Supplementary information

Supplementary Information.
