# Supplementary material for: Serum angiopoietin-like 3 levels are elevated in obese non diabetic men but are unaffected during an oral glucose tolerance test
Source: Sci Rep. 2020 Dec 3;10:21118. doi: 10.1038/s41598-020-77961-8 (PMC7713064; doi:10.1038/s41598-020-77961-8)
Supplement: Supplementary file 1 — Supplementary Information. [file 41598_2020_77961_MOESM1_ESM.docx]

Supplementary Material

Serum Angiopoietin-Like 3 levels are elevated in obese non diabetic men but are unaffected during an oral glucose tolerance test

Maria Fernanda Garcés^1^, Julieth Daniela Buell - Acosta^1^, Haiver Antonio Rodríguez – Navarro^1^, Estefania Pulido -Sánchez^1^, Juan José Rincon - Ramírez^1^, Diana Carolina Moreno - Ordóñez^2^, Roberto Franco – Vega^2^, Jhoan Sebastian Roncancio – Muñoz^3^, Alvaro Javier Burgos - Cardenas^3^, Ezequiel Lacunza^4^, Justo P. Castaño^5^, Carlos Diéguez^6^, Rubén Nogueiras^6^, Elizabeth Sanchez^1^ and Jorge Eduardo Caminos^1^

^1^Department of Physiology, ^2^Endocrine Unit - Department of Internal Medicine and ^3^Department of Internal Medicine, School of Medicine, Universidad Nacional de Colombia, Bogota, Colombia. ^4^CINIBA, Facultad de Ciencias Médicas, Universidad Nacional de La Plata, La Plata, Argentina. ^5^Maimonides Institute of Biomedical Research of Cordoba (IMIBIC); Department of Cell Biology, Physiology and Immunology, Universidad de Córdoba; Reina Sofia University Hospital; and CIBER Physiopathology of Obesity and Nutrition (CIBERobn); Córdoba, Spain.^6^Department of Physiology (CIMUS), School of Medicine, Instituto de Investigaciones Sanitarias (IDIS), Universidad de Santiago de Compostela, Santiago de Compostela, Spain.

**Supplementary Materials:**

**Figure S1.** Relationship between fasting serum ANGPTL3 levels and; (A) Leptin, (B) Leptin/adiponectin index, (C) TyG index (Triglycerides/glucose index). A p-value < 0.05 was considered statistically significant.

**Figure S2.** Relationship between serum ANGPTL3 area under the curve (AUC) and; (A) Body mass index (BMI), (B) waist circumference, (C) Waist/Height ratio, (D) Android fat mass (AFM %), (E) Gynoid fat mass (GFM %), (F) Total body fat (TBF %). A p-value<0.05 was considered statistically significant.

**Figure S3.** Relationship between serum ANGPTL3 area under the curve (AUC) and; (A) Matsuda Index, (B) insulin area under the curve (AUC). A p-value<0.05 was considered statistically significant


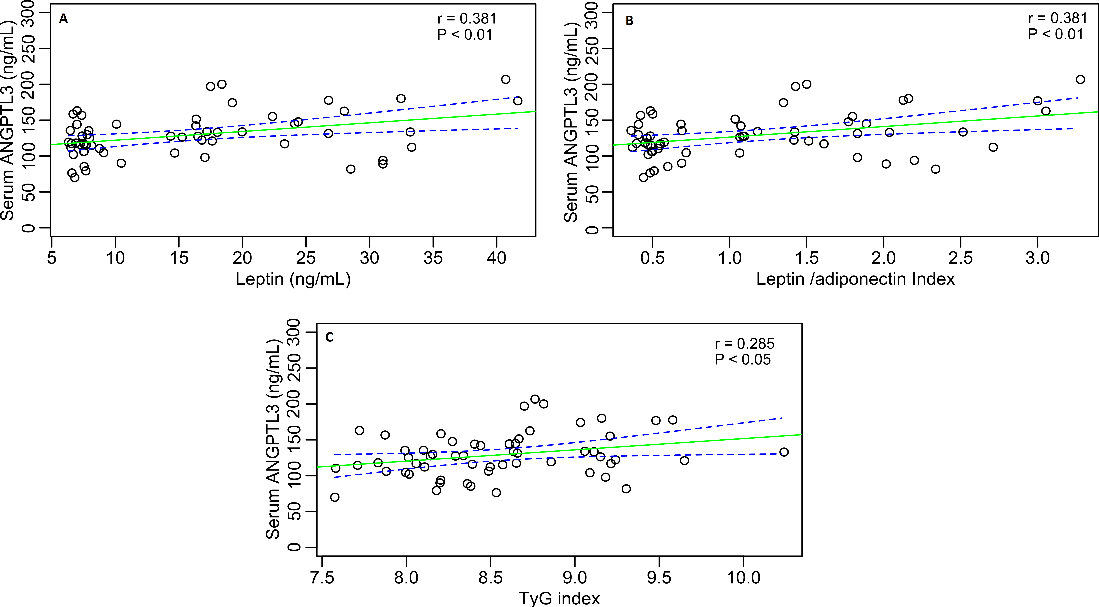
*Supplementary Figure 1*


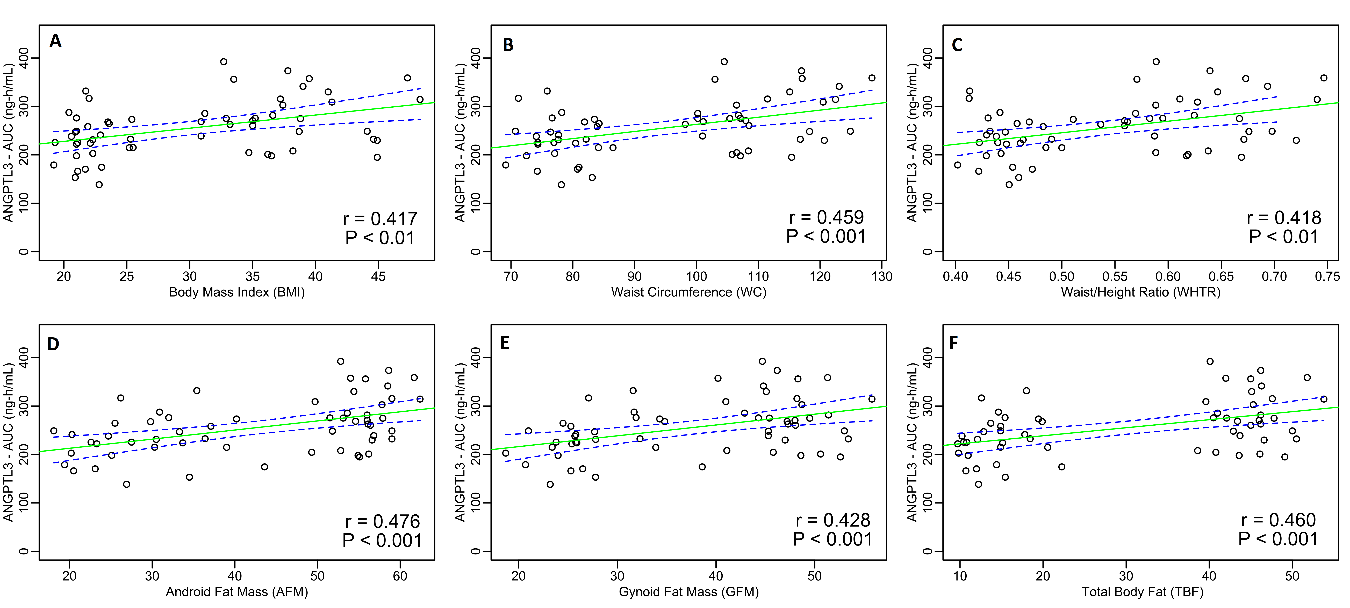


*Supplementary Figure 2*


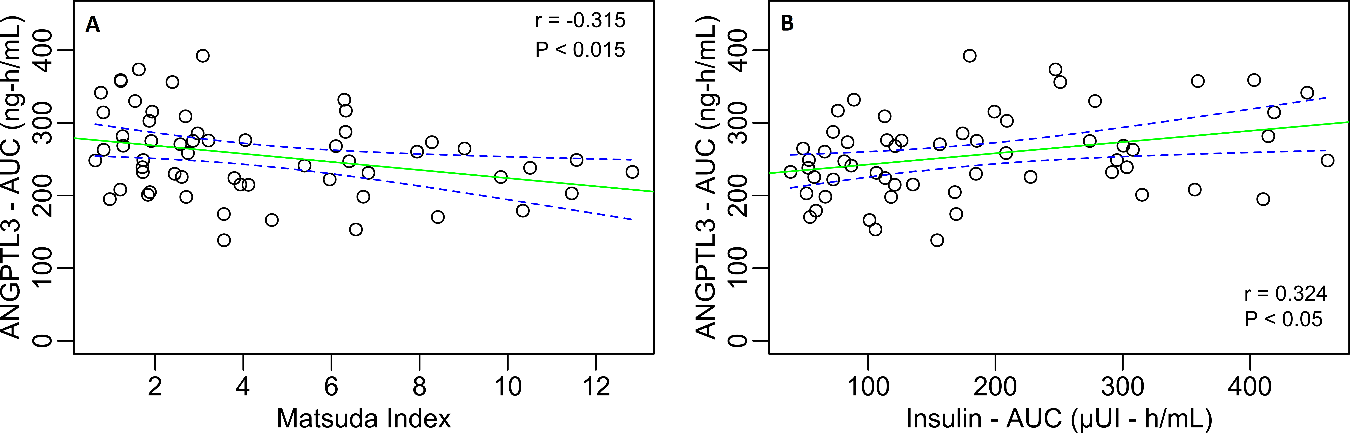


*Supplementary Figure 3*
